# Mutant huntingtin activates Nrf2-responsive genes and impairs dopamine synthesis in a PC12 model of Huntington's disease

**DOI:** 10.1186/1471-2199-9-84

**Published:** 2008-10-09

**Authors:** Willeke MC van Roon-Mom, Barry A Pepers, Peter AC 't Hoen, Carola ACM Verwijmeren, Johan T den Dunnen, Josephine C Dorsman, GertJan B van Ommen

**Affiliations:** 1 Center for Human and Clinical Genetics, Albinusdreef 2, 2333ZC Leiden, the Netherlands; 2 Department of Neurology, Albinusdreef 2, 2333ZC Leiden, the Netherlands; 3 Leiden Genome Technology Center, Leiden University Medical Center, Albinusdreef 2, 2333ZC Leiden, the Netherlands; 4 Department of Clinical Genetics, Vrije Universiteit Medical Center, Van der Boechorststraat 7, 1081 BT Amsterdam, the Netherlands

## Abstract

**Background:**

Huntington's disease is a progressive autosomal dominant neurodegenerative disorder that is caused by a CAG repeat expansion in the HD or Huntington's disease gene. Although micro array studies on patient and animal tissue provide valuable information, the primary effect of mutant huntingtin will inevitably be masked by secondary processes in advanced stages of the disease. Thus, cell models are instrumental to study early, direct effects of mutant huntingtin. mRNA changes were studied in an inducible PC12 model of Huntington's disease, before and after aggregates became visible, to identify groups of genes that could play a role in the early pathology of Huntington's disease.

**Results:**

Before aggregation, up-regulation of gene expression predominated, while after aggregates became visible, down-regulation and up-regulation occurred to the same extent. After aggregates became visible there was a down-regulation of dopamine biosynthesis genes accompanied by down-regulation of dopamine levels in culture, indicating the utility of this model to identify functionally relevant pathways. Furthermore, genes of the anti-oxidant Nrf2-ARE pathway were up-regulated, possibly as a protective mechanism. In parallel, we discovered alterations in genes which may result in increased oxidative stress and damage.

**Conclusion:**

Up-regulation of gene expression may be more important in HD pathology than previously appreciated. In addition, given the pathogenic impact of oxidative stress and neuroinflammation, the Nrf2-ARE signaling pathway constitutes a new attractive therapeutic target for HD.

## Background

Huntington's disease (HD) is a progressive autosomal dominant neurodegenerative disease caused by a CAG repeat expansion in the coding region of the *HD *gene, resulting in an expansion of polyglutamines at the N-terminus of the huntingtin protein and accumulation of the mutant protein into cytoplasmic and nuclear aggregates [1]. Larger expansions result in increased aggregation and an earlier onset of pathological and clinical features [2]. The formation of aggregates is considered a hallmark of HD [3,4]. The neuropathology of HD involves a selective dysfunction and death of specific neuronal subpopulations within the central nervous system [5].

Gene expression studies on animal model material and low grade patient material have provided important information on cellular processes and pathways involved in HD pathology [6,7]. Not only is there a loss of normal huntingtin function [8], upon expansion there is a toxic gain of function resulting in a disruption of cellular functions, including transcriptional deregulation, caspase activation and aberrant proteasomal processing [9]. In addition, an increase in reactive oxygen species was found.

Furthermore, the regional pattern of transcriptional pathology in humans is in agreement with the pattern of neurodegeneration [6]. The first detectable changes in gene expression in mice carrying the first exon of mutant huntingtin coincided with the first occurrence of pathological and behavioural changes [10], again underscoring the fact that gene expression is a good reflection of ongoing changes towards pathology. Furthermore, transcriptional changes, especially in the mouse models reflecting more advanced stages of the disease, were similar to the changes seen in human HD brain [7].

Although micro array studies on patient and animal tissue provide valuable information, the primary effect of mutant huntingtin will inevitably be masked by secondary processes in advanced stages of the disease. Thus, cell models are instrumental to study early, direct effects of mutant huntingtin, offering new insights into the pathogenic mechanisms of HD. Especially studies of the early stage are extremely important, because in this stage the phenotype can still be reverted. A well established HD cell model is the rat phaeochromocytoma (PC) 12 cell line, which inducibly expresses exon 1 of the *HD *gene [11,12]. So far comprehensive expression profiling studies exploiting data of the completed rat genome project were lacking. The aim of this study was to find mRNA changes early in the cellular pathology before and after the occurrence of aggregates and to identify groups of genes that could play a role in the pathology of HD.

## Methods

### Cell culture

Inducible rat PC12 cell lines expressing an exon 1 fragment of huntingtin with 23 (Q23) or 74 (Q74) glutamine repeats fused to the Green Fluorescent Protein (GFP), [11,12] were cultured in standard high glucose Dulbecco's modified Eagle's medium (DMEM, Invitrogen Life Technologies, Carlsbad, USA) supplemented with 100 U/ml penicillin/streptomycin (Invitrogen Life Technologies), 2 mM L-glutamine (Invitrogen Life Technologies), 10% heat-inactivated horse serum (Invitrogen Life Technologies), 5% Tet-approved heat inactivated fetal bovine serum (Clontech, Palo Alto, USA), 100 μg/ml G418 (Invitrogen Life Technologies) and 75 μg/ml hygromycin (Invitrogen Life Technologies) at 37°C and 10% CO_2_. Cells were induced with 1 μg/ml doxycycline (dox, Clontech) and harvested on day 0 (uninduced cells), 1 day (when only a few cells expressing mutant huntingtin contain aggregates) and 5 days (when nearly all cells expressing mutant huntingtin contain aggregates) [12]. The same culture conditions were used for PC12 cells without a construct, to eliminate the effect of doxycycline treatment on gene expression.

### Hybridization design

For each construct, we performed duplicate experiments with 2 independent cell lines for each construct (biological replicates). Furthermore, from each cell line, two separate RNA isolations were performed (technical replicates). RNA was harvested prior to induction (day 0), after 24 hours of induction (day 1) and after 5 days of induction. RNA was isolated with the QIAGEN RNeasy Mini Kit (QIAGEN, Hilden, Germany) with on-column DNase treatment. First-strand and second-strand cDNA synthesis from total RNA was performed and biotin-modified NTPs were incorporated into the *in vitro *transcribed cRNA with the Illumina TotalPrep RNA Amplification Kit (Ambion, Foster City, USA). This biotin modified cRNA was hybridized to the Illumina RatRef-12 Expression BeadChip array (Illumina Inc., San Diego, CA, USA) and labelled with Streptavidin-Cy3.

### Statistical analysis

The average expression value of all beads per probe with no background correction was obtained from BeadStudio 3.1.7. Further data analysis was performed in 'R' version 2.4.1. [13]. Data were normalized with the VSN method using the VSN package for 'R' [14]. To establish significance of differential gene expression, we used the empirical Bayes moderated t-statistics implemented in LIMMA package version 2.8.1. for 'R' [15] and contrasts between Q23 and Q74 were calculated per time point. Results were adjusted for multiple testing by the Benjamini-Hochberg method. Only probes were considered where the ratio on day 0 had a *P*-value of >0.05 and the ratio on day1 or day 5 had a *P*-value of <0.05. Microarray data discussed in this work have been deposited in NCBI's Gene Expression Omnibus (GEO; ) and are accessible through GEO series accession number GSE10581.

### Functional classification

Functional classification was performed with the web based tool DAVID [16]. This objective tool uses the one-tail Fisher Exact Probability Value for gene-enrichment analysis. Significant gene ontology terms for the Biological Process (BP), Cellular Component (CC) and Molecular Function (MF) ontologies were analysed. Transcripts that showed a significant increased or decreased differential expression at 1 day and 5 days were analyzed separately against a background list of all genes present on the platform. Only categories with a *P*-value < 0.05 containing two or more genes were considered.

### Quantitative RT-PCR

Isolated total RNA, as described above, was also used for qRT-PCR. RNA was quantified spectrometrically and integrity was checked with the Agilent 6000 Nano Assay (Agilent, Santa Clara, USA). Reverse transcription of RNA was performed with the Transcriptor First Strand cDNA Synthesis Kit (Roche, Basel, Switzerland). First Strand cDNA Synthesis was performed with 2 μg of total RNA and 2 μl (600 pmol/μl) random hexamer primers.

The LightCycler 480 PCR and detection system (Roche) was used for amplification and real-time quantification. PCR reactions of each sample were performed in triplicate in a final volume of 10 μl in a 384 well plate (for primer sequences see Additional File 1). The PCR mixture contained 2 μl of 50× diluted cDNA template, 2 μl of 5× qPCR-&GO LC480 Green Mastermix (Roche) (with 12.5 mM MgCl_2_) and primers at a final concentration of 250 nM with the following conditions: initial denaturation at 95°C for 10 min, followed by 45 amplification cycles at 95°C for 10 s, 60°C for 30 s, and 72°C for 20 s and final extension at 72°C for 5 min. After the amplification reaction melting curve analysis was performed, starting at 60°C and increasing to 98°C with 5 acquisitions/°C. Relative mRNA levels were calculated with the efficiency-corrected Ct method with β-actin and Ywhaz as reference genes and mRNA levels on day 0 (uninduced cells) as calibrator [17].

### Immunohistochemistry

Cells were fixed in 4% paraformaldehyde for 15 min and permeabilized in 50% methanol and 1% H_2_O_2 _for 20 min. After three washes in PBS containing 0.2% Triton X-100 (PBS-Triton), cells were incubated overnight at 4°C with rabbit anti tyrosine hydroxylase (1:2000, Santa Cruz Biotechnology Inc., USA) in PBS-Triton with 1% normal goat serum and 0.4% Thimerosal (Sigma, St. Louis, USA). Cells were subsequently incubated with rabbit anti Alexa 594 (Invitrogen Life Technologies) and examined on a Leica TCS SL confocal microscope.

### Western blotting

Whole cell extracts (25 μg per lane) were separated by SDS-PAGE and transferred to a PVDF membrane (Roche). Blots were labelled with rabbit anti Tyrosine Hydroxylase 1:2000 (sc-14007, Santa Cruz Biotechnology Inc., Santa Cruz, USA) followed by goat anti rabbit IgG conjugated to horseradish peroxidase (Santa Cruz) in a 1:10,000 dilution and visualized by ECL plus (Amersham, Buckinghamshire, United Kingdom). Blots were stripped by incubating in stripping buffer (100 mM β-mercapto-ethanol, 2% SDS, 62.5 mM Tris-HCl pH 8.0) for 30 min at 55°C and subsequently labelled for rabbit anti actin antibody 1:600 (clone C4, ICN Biochemicals Inc., Costa Mesa, USA) and goat anti rabbit IgG conjugated to horseradish peroxidase (Santa Cruz) to check that equal amounts of protein were loaded.

### High Performance Liquid Chromatography (HPLC)

Whole cell extracts were prepared as described in the Western blotting paragraph for HPLC analysis. Extracts from 2 independent cell lines for each construct were frozen immediately at -20°C until further analysis. Dopamine concentration was measured by HPLC technology with electron capture detection with a detection limit of 0.01 nmol/L [18]. Dopamine concentrations are represented as μmol dopamine per gram of protein extract.

## Results

On day 1 after doxycycline-mediated induction of the expression constructs, when aggregates had just started to form, there were 145 of the approximately 22,500 transcripts with a significantly changed ratio between Q23 and Q74 (*P *< 0.05 after correction for multiple testing). Of those, 97 showed an increased expression compared to the expression level on day 0, and 48 showed a decreased expression. After 5 days of induction, when there was extensive aggregation of the Q74 but not the Q23 containing protein, there were 1233 transcripts differentially expressed of which 573 transcripts were up and 660 were down. Of the 145 differentially expressed transcripts at day 1, there were 51 also differentially expressed at day 5, while 8 could be expected by chance alone. The other 95 returned to control levels at day 5.

### Cellular processes changed before formation of aggregates

We were particularly interested in the molecular pathways changed in the early stages of HD. Therefore, we evaluated which pathways were represented in the list of transcripts with differential expression at day 1. Since there were no significant categories when GO-based functional classification was performed, all significantly changed transcripts at 1 day were grouped according to their function (see Table 1). Out of the 97 up-regulated transcripts, 54 were included in the analysis while the remaining 43 transcripts were of unknown function. Out of the 48 down-regulated transcripts, 39 were analyzed and there were 9 transcripts of unknown function. The largest functional group consisted of up-regulated transcripts coding for ion channels and receptors, with an over-representation of olfactory receptors. The next two largest groups contained transcriptional and chromatin remodelling related transcripts that were both up- and down-regulated. Transcripts involved in cytoskeletal organization and cellular matrix were mostly down-regulated in the mutant cells and there seemed to be early signs of oxidative stress and impairment in vesicle trafficking.

**Table 1:** Functional grouping of genes that show a significant change in expression between mutant and wild-type cells (Q74-Q23) after 1 day of induction

***Genes up after 1 day of mutant huntingtin expression***				***Genes down after 1 day of mutant huntingtin expression***			
	
*Gene*	*Accession #*	*Gene*	*Accession #*	*Gene*	*Accession #*	*Gene*	*Accession #*
**Apoptosis-related proteins**				**Cell cycle and growth factors**			
Gstp1	XM_579338	Tnfaip8*	XM_225940	Igf2	NM_031511	Cdc14a*	XM_227618
**Cell cycle and growth factors**				Smpd3	NM_053605		
Tgfa	NM_012671			**Cell signalling**			
**Cell signalling**				Arl6*	XM_344009	Plxnb1*	XM_236640
Flrt2*	XM_234361	Rgs14	NM_053764	Calm1	XM_579543	Panx2	XM_579735
Rasl11b	NM_001002830	GAP1(sim)	XM_227132	**Chromatin remodeling – transcription**			
Rgs2	NM_053453			Smad4	NM_019275	Cbx6*	XM_576309
**Chromatin remodeling – transcription**				Chc1l	NM_199084	Ttf2*	XM_215670
Hoxb13*	XM_220905	Vax2	NM_022637	Prickle1	XM_235609	Dnajb6*	XM_342607
LOC362865	XM_343194	LOC368057	XM_347220	Mdc1	XM_227971		
Npas1*	XM_238770	Dclre1b*	XM_227537	**Cytoskeleton – cellular matrix**			
Hoxb9*	XM_220887			Epb4.1l4a*	XM_226060	RGD1310323	NM_001008348
**Cytoskeleton – cellular matrix**				Add3	NM_031552	Cilp*	XM_236348
Mybph	NM_031813			Plekhh1*	XM_234332		
**Electron transport – mitochondrion**				**Electron transport – mitochondrion**			
LOC293949	XM_215248			Dhrs4	NM_153315		
**Ion channels – receptors**				**Ion channels – receptors**			
Ms4a8b*	XM_342026	Olr677*	NM_001001062	Boc*	XM_340986	Ms4a11*	XM_342028
Ccr7	NM_199489	Olr1340*	XM_236174	LOC501049	XM_576464	Olfml2b*	XM_222868
Trpv6	NM_053686	Olr1597*	NM_001000911	Trpv2	NM_017207		
Olr29*	NM_001000691	Ttyh3*	XM_221962	**Oxidative stress**			
Olr552*	NM_001001055	Il17rb*	XM_224604	Prdx6	NM_053576		
Olr1751*	NM_001000492			**RNA processing and ribosomal**			
**Oxidative stress**				Mrps15	NM_001007653	Rps16	XM_341815
Blvra	NM_053850	Indo	NM_023973	**Vesicle trafficking**			
**RNA processing and ribosomal**				Sec24d*	XM_227663	Sytl4	NM_080410
Ear11	NM_138902	Rps24	NM_031112	**Other**			
Trim21*	XM_219011	Ptrh1*	XM_342416	Tekt2*	XM_575908	LOC500847	XM_576239
**Vesicle trafficking**				Chst1*	XM_575178	Adhfe1*	XM_342794
Eml1*	XM_343109			Lypla2	NM_031342	Pigl	NM_138901
**Other**				LOC365544	XM_345087	Herpud1	NM_053523
LOC499985	XM_575339	Atad2*	XM_235326	LOC367880	XM_579653		
Smyd2	XM_213972	LOC498435	XM_573687				
Tpbg	NM_031807	Smpx	NM_053395				
C4.4a	NM_021759	Mocs1*	XM_236911				
Abhd1	NM_001008520	Serpinf1	NM_177927				
Ppic	NM_001004215	Smp2a	NM_012695				
Aak1*	XM_232172	Rhcg	NM_183053				
Prss8	NM_138836	Prlph	NM_021580				
LOC367332	XM_346086	Xylt1	XM_341912				
Cyp4a10	NM_153307						

* predicted

### Cellular processes changed after formation of aggregates

Results of the GO-based functional classification of transcripts with differential expression between Q23 and Q74 expressing cells 5 days after induction, are presented in Table 2. Only functional categories that were significantly overrepresented (*P *< 0.05; hypergeometric test) compared to the full list of features present on the array are displayed. The most significant terms for the up-regulated transcripts were in the Biological Process (BP) category: negative regulation of dendrite morphogenesis and fibroblast proliferation, for the Cellular Component (CC) category: antioxidant activity and stress fiber, and for the Molecular Function (MF) category: electron transport activity and chemokine receptor binding. The most significant terms for the down-regulated transcripts were in the BP category: regulation of heart contraction and isoprenoid biosynthesis, for the CC category: endosome membrane and actin cytoskeleton, and for the MF category: double-stranded DNA binding and SH3/SH2 adaptor activity. Apoptosis related transcripts were expressed at higher levels in the Q74 cells, as were transcripts involved in mitochondrial biogenesis and electron transporter activity, and transcripts involved in antioxidant activity. This indicates an increased mitochondrial activity and a response to oxidative stress in our cell model that was already suggested at day 1. Actin and cytoskeletal-related transcripts were mostly decreased in expression in the mutant cell line compared to the wild-type cell line and there was a large transcription regulation component in the down-regulated transcripts. Cholesterol metabolism was also significant in the group of down-regulated transcripts, which is a pathway that was found in earlier gene expression studies in HD [19].

**Table 2:** Functional classification analysis of genes changed significantly after 5 days of mutant huntingtin expression

**Functional Classification with genes that showed an increased expression after 5 days**				**Functional Classification with genes that showed a decreased expression after 5 days**			
*Ontology*	*Term*	*# genes*	*P value*	*Ontology*	*Term*	*# genes*	*P value*
BP	Negative regulation of dendrite morphogenesis	3	0,004904	BP	Regulation of heart contraction	7	0,000823
	Negative regulation of fibroblast proliferation	3	0,004904		Isoprenoid biosynthesis	4	0,003744
	Cell adhesion	16	0,009781		Galactose metabolism	3	0,005365
	Response to DNA damage stimulus	8	0,011782		Angiogenesis	9	0,007585
	Positive regulation of caspase activity	4	0,014506		Response to stress	40	0,007784
	Positive regulation of hydrolase activity	4	0,019419		Neg. regulation of transcription from RNA polII promoter	8	0,007866
	Apoptotic nuclear changes	3	0,021186		Cell motility	18	0,008263
	Mitochondrial membrane organization and biogenesis	3	0,021186		Pos. regulation of transcription from RNA polII promoter	10	0,010333
	Metal ion homeostasis	8	0,021801		Actin cytoskeleton organization and biogenesis	10	0,011951
	Carbohydrate biosynthesis	6	0,023170		Blood vessel morphogenesis	9	0,015755
	Biopolymer metabolism	38	0,024409		Cholesterol metabolism	7	0,016064
	Fibroblast proliferation	3	0,026721		Glucose catabolism	6	0,022220
	Protein oligomerization	4	0,035235		Histogenesis and organogenesis	3	0,024619
CC	Antioxidant activity	5	0,015651		Regulation of neuronal synaptic plasticity	4	0,029030
	Stress fiber	3	0,017610		Somitogenesis	3	0,033500
	Cytoplasmic membrane-bound vesicle	10	0,019229		Synaptic transmission, dopaminergic	3	0,033500
	Nuclear chromosome	4	0,030424		cAMP metabolism	4	0,039722
MF	Electron transporter activity	11	0,001023		Neurotransmitter transport	6	0,046079
	Chemokine receptor binding	6	0,001069	CC	Endosome membrane	3	0,008890
	Metalloendopeptidase inhibitor activity	3	0,007766		Actin cytoskeleton	9	0,033881
	Selenium binding	4	0,008247		Cell projection	13	0,033985
	Protease inhibitor activity	7	0,013043		Cytoplasmic membrane-bound vesicle	12	0,038151
	GTPase activity	8	0,013763		Mitochondrion	24	0,043218
	Guanyl nucleotide binding	9	0,040251		Spindle	4	0,048198
	Zinc ion binding	17	0,041022	MF	Double-stranded DNA binding	7	0,001838
244 genes included in analysis					SH3/SH2 adaptor activity	5	0,004637
					Oxidoreductase activity, acting on the CH-CH group of donors	6	0,004683
					Cell cycle regulator	4	0,005046
					Epidermal growth factor receptor binding	3	0,023409
					Protein phosphorylated amino acid binding	3	0,023409
					Actin binding	10	0,026446
					Monoamine transporter activity	3	0,031878
					SNAP receptor activity	3	0,031878
					Carbohydrate kinase activity	4	0,031902
					Transcriptional activator activity	11	0,045287
				331 genes included in analysis			

BP: Biological Process.

CC: Cellular Component.

MF: Molecular Function

Besides these significantly changed GO terms, a closer examination of the array data revealed 5 transcripts of the TGF beta signalling pathway significantly down-regulated after 5 days (see Table 3) with the TGF beta transducer Smad4 already down-regulated after 1 day of mutant huntingtin expression. Two out of the eight Smad proteins involved in the TGF beta signalling pathway were down-regulated as well as TGF beta1.

**Table 3:** Oxidative stress related genes that show a significant change with microarray analysis after mutant huntingtin expression

**Nrf2 responsive genes**								
*Gene name*	*Accession#*	*Ratio day 0*	*Ratio day 1*	*Ratio day 5*	*P value day 0*	*P value day 1*	*P value day 5*	
***Detoxification***								
Nqo1	NM_017000	0,153161338	0,09509419	0,9313329	0,833712475	0,927082425	0,000223519	▲
Gsta4	XM_217195	-0,275666227	-0,314801025	0,413131618	0,253015701	0,120614239	0,006374434	▲
Gstp2	NM_138974	0,382499361	0,418277366	1,459294326	0,30465786	0,189513652	2,28E-07	▲
***Antioxidant/reducing Potential***								
Gclc	NM_012815	0,050889982	0,064489288	0,553224444	0,935159305	0,921933889	0,000668569	▲
Txnrd1	NM_031614	0,043006071	0,068186563	0,416963683	0,920763898	0,85987218	0,000404461	▲
Prdx6	NM_053576	-0,529339334	-0,578113241	-0,680873497	0,084587704	0,038158555	0,003140611	▼
Taldo1	NM_031811	0,009418008	0,032634451	-0,237774271	0,986167462	0,947870016	0,026954523	▼
Me1	NM_012600	-0,000528861	0,013155505	0,269261838	0,998825398	0,98787689	0,021535996	▲
								
*Gene name*	*Accession#*	*Ratio day 0*	*Ratio day 1*	*Ratio day 5*	*P value day 0*	*P value day 1*	*P value day 5*	

***TGF beta signalling pathway***								
Bambi	NM_139082	-0,039617538	-0,026338592	-0,864880442	0,977785277	0,990188964	0,00336128	▼
Smad4	NM_019275	-0,203348936	-0,40795009	-0,373068895	0,356782837	0,006017924	0,003289649	▼
Map3k1	NM_053887	-0,299368715	-0,358856734	-0,690663403	0,55056724	0,382654757	0,004166232	▼
Tgfb1	NM_021578	-0,126020083	-0,168731098	-0,366913617	0,684930689	0,490885028	0,004166232	▼
Madh7 (smad7)	NM_030858	-0,264074403	-0,230874139	-0,588447764	0,720614387	0,784042896	0,049193592	▼
***Oxidative stress***								
Epas1	NM_023090	-0,270174359	-0,478814403	-1,093139357	0,688153639	0,259648489	0,000173015	▼
Frd1	XM_574642	0,131200174	0,166324378	0,387371459	0,560092481	0,355116774	0,000497262	▲
Mt3	NM_053968	0,415104171	0,340566391	1,552629091	0,456685437	0,588382185	4,64E-06	▲
Gpx1	NM_030826	0,118639332	0,007736138	0,307368335	0,5834878	0,991968688	0,002399689	▲
***Electron transport activity***								
Nqo1	NM_017000	0,153161338	0,09509419	0,9313329	0,833712475	0,927082425	0,000223519	▲
Cyb561	XM_221030	-0,028644328	-0,034319208	-0,617267222	0,977340962	0,978641469	0,003179802	▼
Cxcl10	NM_139089	0,113398034	0,222659258	1,175083864	0,855674156	0,616446381	1,06E-06	▲
Phgdh	NM_031620	0,019449285	0,127790463	0,21275382	0,963270227	0,538281599	0,044674848	▲
Akr1b4	NM_012498	0,211752133	0,205849841	0,441397541	0,661683708	0,680952818	0,037660927	▲

▲: increased expression; ▼: decreased expression; underscore means *P *value<0.05

### Dopamine biosynthesis critically affected

**Figure 1:**
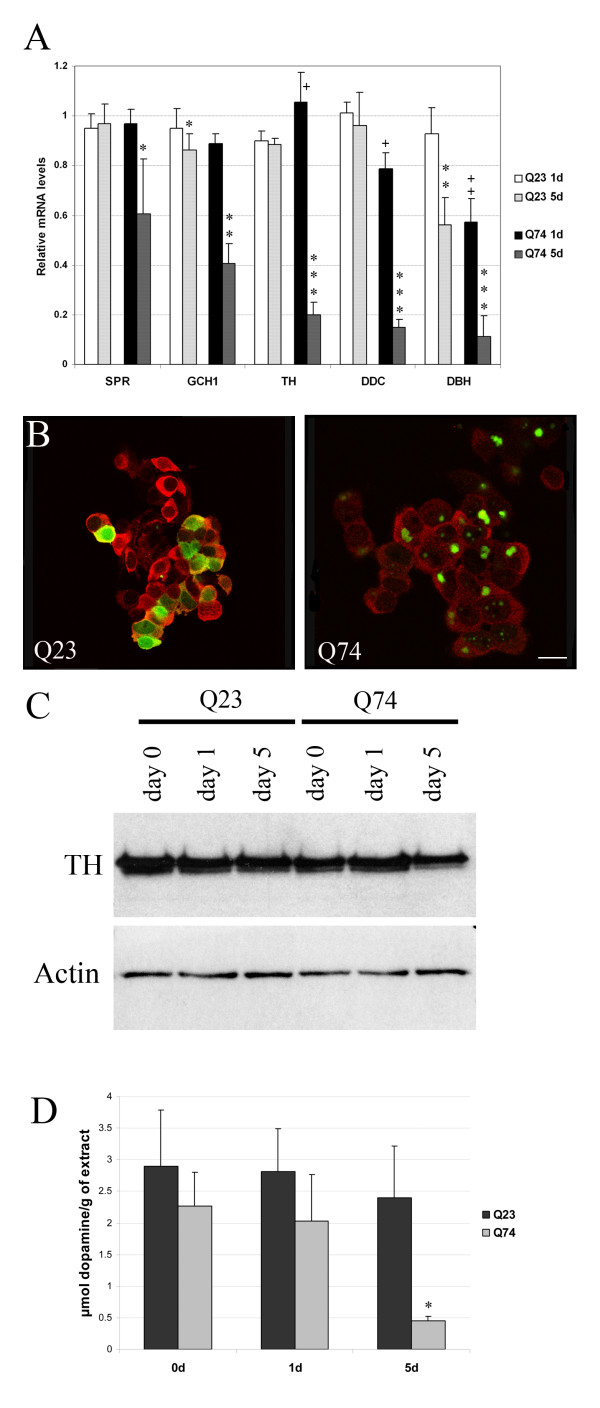
**Down regulation of the dopamine biosynthesis pathway in the PC12 model of Huntington's disease**. **A**: qRT-PCR was performed on samples from Q 23 and Q74 cells for 0, 1 and 5 days. All values are expressed as relative mRNA levels compared to expression levels of uninduced cells. SPR: Sepiapterin reductase, GCH1: GTP cyclohydroxylase 1, TH: Tyrosine hydroxylase, DDC: Dopa decarboxylase, DBH: Dopamine β-hydroxylase. * Significant differences between the day 1 and 5 days; * *P *< 0.05, **P < 0.005, ***P < 0.0005 student's t-test. Significant differences between Q23 on day 1 and Q74 on day 1; + *P *< 0.05, ++*P *< 0.005 student's t-test. **B**: Confocal microscopic images of Q23 and Q74 PC12 cells after 5 days of induction. Green represents huntingtin-GFP and red represents TH immunohistochemistry. In Q23 cells, both huntingtin and TH show a diffuse staining, while in Q74 cells, large aggregates are visible and a clear reduction of TH fluorescence. Scale bar = 25 μm. **C**: Western blot analysis on whole cell lysates of Q23 and Q74 cells with TH and β-actin as loading control. A clear reduction in TH level can be seen in the 5 day Q74 samples. **D**: HPLC measurements in whole cell lysates of Q23 and Q74 cells after 0, 1 and 5 days of huntingtin expression show a significant reduction in dopamine levels in the 5 day Q74 samples. (* *P *< 0.05, student's t-test).

Further examination of the dopamine biosynthesis pathway by qRT-PCR revealed 5 genes involved in the dopamine biosynthesis expressed at lower levels in the mutant cell line compared to the wild-type cells (see Figure 1A).

To analyze the effect of these mRNA changes on protein level, the TH protein was examined by immunohistochemistry on cultured cells revealing a diffuse cytoplasmic localization in the cells expressing wild type and mutant huntingtin but there was a marked decrease of TH fluorescence in mutant cells (see Figure 1B). This reduction in TH was confirmed by Western blotting of cell lysates from independent isolations and a representative result is shown in Figure 1C. This decrease in TH resulted in a significant decrease in dopamine levels after 5 days in the cells expressing mutant huntingtin as measured by HPLC (see Figure 1D).

### Oxidative stress

Many oxidative stress-related transcripts were up-regulated and several classic Nrf2 (NF-E2 related factor 2/Nfe2l2) responsive transcripts and oxidative stress related genes (see Table 3) were differentially expressed after analysis of the gene expression array.

**Figure 2:**
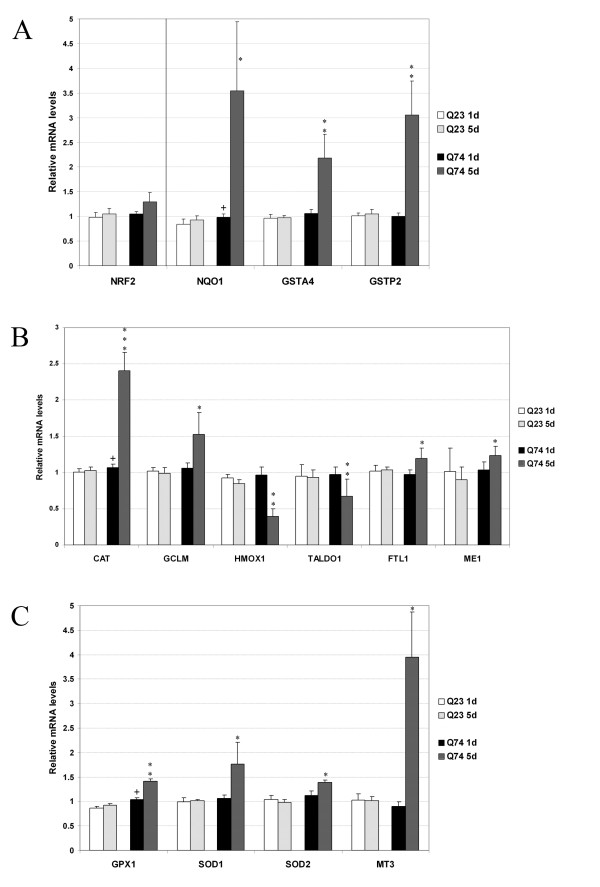
**Nrf2-responsive genes and oxidative stress related genes were examined by quantitative real-time PCR on samples from cells expressing wild type (Q23) and mutant (Q74) huntingtin for 0, 1 and 5 days**. All values are expressed as relative mRNA levels compared to expression levels on day 0 (uninduced cells) with β-actin and Ywhaz as reference genes. **A**: Nrf2 and Nrf2-responisve genes involved in detoxification. NRF2: NF-E2 related factor 2, NQO1: NAD(P)H dehydrogenase quinone 1, GSTA4: glutathione S-transferase alpha 4, GSTP2: glutathione S-transferase pi2. **B**: Nrf2-responsive genes involved in antioxidant/reducing potential. CAT: catalase, GCLC: the catalytic subunit of glutamate-cysteine ligase, GCLM: the modifier subunit of glutamate-cysteine ligase, HMOX1: heme oxygenase 1, TALDO1: transaldolase 1, FTL1: ferritin light chain, Me1: malic enzyme 1. **C**: Non-Nrf2 responsive genes involved in protection against oxidative stress. GPX1: glutathione peroxidase, SOD1: superoxide dismutase 1, SOD2: superoxide dismutase 2, MT3: metallothionein 3. * Represents significant differences between the day 1 and day 5 time points (* *P *< 0.05, **P < 0.005, ***P < 0.0005 student's t-test). + Represents significant differences.

Nrf2-responsive genes have been identified in previous studies in cultures of primary cortical astrocytes and primary neuronal cultures and were classified as genes involved in the antioxidant and detoxification response, transcription, growth, inflammation and signalling pathways [20-22]. Interestingly, most Nrf2-responsive transcripts involved in detoxification and antioxidant/reducing potential were increased in expression in the present study, while Nrf2-responsive transcripts involved in transcription, growth, inflammation and signalling pathways were not changed or expressed at lower levels in the mutant cells. Subsequently, Nrf2-responsive genes involved in antioxidant/reducing potential as well as some non-Nrf2 responsive genes involved in protection against oxidative stress were further examined by qRT-PCR analysis. This analysis included additional oxidative stress related genes that were present on the microarray but where the change in gene expression did not reach statistical significance. Increased Nrf2-responsive transcripts that have a protective role in the metabolic detoxification of reactive intermediates are shown in Figure 2A. Nrf2-responsive genes that act as antioxidants and are reducing agents that prevent oxidative reactions that were expressed at higher levels in the mutant cell line are shown in Figure 2B. While most Nrf2-responsive genes were up-regulated, Hmox1, Taldo1 and Prdx6 were down-regulated.

Other transcripts, not identified as Nrf2- target genes, but important antioxidant enzymes involved in the protection against oxidative stress, that were also expressed at higher levels in the mutant cell line are shown in Figure 2C. Finally, several transcripts involved with electron transport activity were expressed at higher levels on the array in the cells expressing mutant huntingtin (see Table 3).

## Discussion

To identify groups of genes that could play a role in the pathology of HD, we studied mRNA changes in an inducible PC12 HD model before and after aggregates became visible. GO term analysis revealed many similarities with previous human and whole animal gene expression studies indicating that this model system is useful to study pathological markers for HD [6,7,23,24]. This is the first study to show the involvement of Nrf2-responsive genes in the oxidative stress response in HD. Oxidative stress related transcripts were altered in expression, suggesting a protective response in cells expressing mutant huntingtin at an early stage of cellular pathology. Furthermore, there was a down-regulation of dopamine biosynthesis resulting in lower dopamine levels in culture. Our results further demonstrate an early impairment of transcription, the cytoskeleton, ion channels and receptors.

Since it is not feasible to study early mRNA changes in humans and it is more difficult to detect mRNA changes in early stage mouse models of HD compared to later stages [10], cell models are important to study the early pathological cellular events in HD. Although cell models are a simplified representation of the disease, it is less likely that pathological markers are masked by secondary disease processes and heterogeneous cell populations. Notably, in the present study, the majority of transcripts that changed on day 1 showed an increase in expression, which is in contrast with previous studies where it was found that most transcripts were decreased in expression at the early stages in a mouse model of HD [7,23,24]. After 5 days of mutant huntingtin expression, more transcripts were significantly changed compared to 1 day, and the ratio of transcripts that increased to transcripts that decreased in expression was approximately 1:1, which is in agreement with previous findings [7]. This suggests that up-regulation of transcription may be more important in early HD pathology than previously appreciated. The relevance of studies on mRNA level for the identification of changes in biological processes is demonstrated for the dopamine biosynthesis pathway, for which we showed that a decrease in mRNA levels leads to concomitant changes in dopamine levels.

We found early signs of oxidative stress with a differential expression of mitochondrial, electron transport and oxidative stress related transcripts. The oxidative stress-response became more obvious after 5 days where transcripts of the Nrf2-ARE pathway were expressed at higher levels in the cells expressing mutant huntingtin. Genes containing the antioxidant response element (ARE) can be activated by Nrf2 in response to oxidative stress [25] in what is called the antioxidant response pathway [26].

Although there has been extensive evidence for a role of oxidative stress early in HD pathogenesis [27] and an age-dependent induction of compensatory mechanisms in response to oxidative stress has been found in the R6/2 mouse model of HD[28], this is the first study implicating Nrf2-responsive genes. While the majority of Nrf2-dependent transcripts increased in expression after the induction of mutant huntingtin, there were two transcripts that showed a validated decreased expression. Thus, only a subset of Nrf2 responsive genes is altered, possibly due to a different combination of transcription factor binding sites [29].

Mitochondrial dysfunction possibly contributes to this oxidative stress, since this is thought to lead to decreased production of ATP and an increase in reactive oxygen species (ROS), ultimately leading to cell damage and cell death [30]. A defect in mitochondrial complexes as well as an increase in oxidative damage products has been found in previous studies [28,31,32] and is in agreement with our findings of an increase in transcripts involved in electron transport activity and oxidative stress response in cells expressing mutant huntingtin.

We also found that the DNA repair gene Transaldolase1, which promotes DNA stability and repair [33], was expressed at lower levels in mutant cells compared to wild-type cells in the current study. This is in agreement with increased DNA oxidative damage products that have been found in post mortem HD brain tissue [34,35].

That activation of the Nrf2 pathway can ameliorate the effects of oxidative stress was shown when induction of Nrf2-mediated transcription could protect cells from mitochondrial complex II inhibition [36] and defects in complex II have been implicated in HD [34,37,38]. Indeed, a study using an *in vitro *model of PD showed that activation of the Nrf2-ARE pathway protected against 6-hydroxydopamine neurotoxicity [39].

## Conclusion

Combined micro array studies thus provide ample evidence for the induction of a protective oxidative stress response in cells expressing mutant huntingtin in an early stage of the pathology [27]. This response may ultimately be insufficient to protect cells from oxidative damage, contributing to the massive cell death seen in HD brains. Oxidative stress is thought to play a role in the pathogenesis of several other neurodegenerative diseases [40] and Nrf2 has been implicated in the pathology of Alzheimer's and Parkinson's disease. Given the pathogenic impact of oxidative stress and neuroinflammation, the Nrf2-ARE signalling pathway is an attractive therapeutic target for neurodegenerative diseases and warrants further studies.

## Authors' contributions

WvR-M carried out the micro array analysis and drafted the manuscript. BP performed RNA isolations, hybridization to the arrays, Western Blot and cell culture experiments. PH participated in the design of the study and statistical analysis. CV participated in the immunohistochemical analysis and maintenance of the cell lines. JD participated in the design of the study and helped to draft the manuscript. JdD participated in the design and coordination of the study. GvO conceived the study, and participated in its design and coordination. All authors read and approved the final manuscript.

## Supplementary Material

Additional file 1 **Primer sequences for quantitative RT-PCR analysis** Click here for file

## References

[B1] Huntington's disease collaborative research group (1993). A novel gene containing a trinucleotide repeat that is expanded and unstable on Huntington's disease chromosomes. Cell.

[B2] Bates G (2003). Huntingtin aggregation and toxicity in Huntington's disease. Lancet.

[B3] Yang W, Dunlap JR, Andrews RB, Wetzel R (2002). Aggregated polyglutamine peptides delivered to nuclei are toxic to mammalian cells. Hum Mol Genet.

[B4] Arrasate M, Mitra S, Schweitzer ES, Segal MR, Finkbeiner S (2004). Inclusion body formation reduces levels of mutant huntingtin and the risk of neuronal death. Nature.

[B5] Vonsattel J-P, Myers RH, Stevens TJ, Ferrante RJ, Bird ED, Richardson EP (1985). Neuropathological classification of Huntington's disease. J Neuropathol Exp Neurol.

[B6] Hodges A, Strand AD, Aragaki AK, Kuhn A, Sengstag T, Hughes G, Elliston LA, Hartog C, Goldstein DR, Thu D (2006). Regional and cellular gene expression changes in human Huntington's disease brain. Hum Mol Genet.

[B7] Kuhn A, Goldstein DR, Hodges A, Strand AD, Sengstag T, Kooperberg C, Becanovic K, Pouladi MA, Sathasivam K, Cha JH (2007). Mutant huntingtin's effects on striatal gene expression in mice recapitulate changes observed in human Huntington's disease brain and do not differ with mutant huntingtin length or wild-type huntingtin dosage. Hum Mol Genet.

[B8] Cattaneo E, Rigamonti D, Goffredo D, Zuccato C, Squitieri F, Sipione S (2001). Loss of normal huntingtin function: new developments in Huntington's disease research. Trends in Neurosciences.

[B9] Rubinsztein DC, Carmichael J (2003). Huntington's disease: molecular basis of neurodegeneration. Expert Rev Mol Med.

[B10] Luthi-Carter R, Hanson SA, Strand AD, Bergstrom DA, Chun W, Peters NL, Woods AM, Chan EY, Kooperberg C, Krainc D (2002). Dysregulation of gene expression in the R6/2 model of polyglutamine disease: parallell changes in muscle and brain. Hum Mol Genet.

[B11] Wyttenbach A, Swartz J, Kita H, Thykjaer T, Carmichael J, Bradley J, Brown R, Maxwell M, Schapira A, Orntoft TF (2001). Polyglutamine expansions cause decreased CRE-mediated transcription and early gene expression changes prior to cell death in an inducible cell model of Huntington's disease. Hum Mol Genet.

[B12] Cong SY, Pepers BA, Evert BO, Rubinsztein DC, Roos RA, van Ommen GJ, Dorsman JC (2005). Mutant huntingtin represses CBP, but not p300, by binding and protein degradation. Mol Cell Neurosci.

[B13] R Development Core Team R: A language and environment for statistical computing.

[B14] Huber W, von Heydebreck A, Sultmann H, Poustka A, Vingron M (2002). Variance stabilization applied to microarray data calibration and to the quantification of differential expression. Bioinformatics.

[B15] Smyth GK, Gentleman R, Dudoit S, Irizarry R, Huber W (2005). Limma: linear models for microarray data. Bioinformatics and Computational Biology Solutions using R and Bioconductor.

[B16] Dennis G, Sherman BT, Hosack DA, Yang J, Gao W, Lane HC, Lempicki RA (2003). DAVID: Database for Annotation, Visualization, and Integrated Discovery. Genome Biol.

[B17] Pfaffl MW (2001). A new mathematical model for relative quantification in real-time RT-PCR. Nucleic Acids Res.

[B18] Graham PE, Smythe GA, Edwards GA, Lazarus L (1993). Laboratory diagnosis of phaeochromocytoma: which analytes should we measure?. Ann Clin Biochem.

[B19] Valenza M, Rigamonti D, Goffredo D, Zuccato C, Fenu S, Jamot L, Strand A, Tarditi A, Woodman B, Racchi M (2005). Dysfunction of the cholesterol biosynthetic pathway in Huntington's disease. J Neurosci.

[B20] Lee JM, Li J, Johnson DA, Stein TD, Kraft AD, Calkins MJ, Jakel RJ, Johnson JA (2005). Nrf2, a multi-organ protector?. FASEB J.

[B21] Lee JM, Calkins MJ, Chan K, Kan YW, Johnson JA (2003). Identification of the NF-E2-related factor-2-dependent genes conferring protection against oxidative stress in primary cortical astrocytes using oligonucleotide microarray analysis. J Biol Chem.

[B22] Lee JM, Shih AY, Murphy TH, Johnson JA (2003). NF-E2-related factor-2 mediates neuroprotection against mitochondrial complex I inhibitors and increased concentrations of intracellular calcium in primary cortical neurons. J Biol Chem.

[B23] Luthi-Carter R, Strand A, Peters NL, Solano SM, Hollingsworth ZR, Menon AS, Frey AS, Spektor BS, Penney EB, Schilling G (2000). Decreased expression of striatal signaling genes in a mouse model of Huntington's disease. Hum Mol Genet.

[B24] Sipione S, Rigamonti D, Valenza M, Zuccato C, Conti L, Pritchard J, Kooperberg C, Olson JM, Cattaneo E (2002). Early transcriptional profiles in huntingtin-inducible striatal cells by microaray analyses. Hum Mol Genet.

[B25] Itoh K, Chiba T, Takahashi S, Ishii T, Igarashi K, Katoh Y, Oyake T, Hayashi N, Satoh K, Hatayama I (1997). An Nrf2/small Maf heterodimer mediates the induction of phase II detoxifying enzyme genes through antioxidant response elements. Biochem Biophys Res Commun.

[B26] Nguyen T, Sherratt PJ, Nioi P, Yang CS, Pickett CB (2005). Nrf2 Controls Constitutive and Inducible Expression of ARE-driven Genes through a Dynamic Pathway Involving Nucleocytoplasmic Shuttling by Keap1. J Biol Chem.

[B27] Browne SE, Beal MF (2006). Oxidative damage in Huntington's disease pathogenesis. Antioxid Redox Signal.

[B28] Fox JH, Barber DS, Singh B, Zucker B, Swindell MK, Norflus F, Buzescu R, Chopra R, Ferrante RJ, Kazantsev A (2004). Cystamine increases l-cysteine levels in Huntington's disease transgenic mouse brain and in a PC12 model of polyglutamine aggregation. J Neurochem.

[B29] Zhang J, Ohta T, Maruyama A, Hosoya T, Nishikawa K, Maher JM, Shibahara S, Itoh K, Yamamoto M (2006). BRG1 interacts with Nrf2 to selectively mediate HO-1 induction in response to oxidative stress. Mol Cell Biol.

[B30] Calabrese V, Scapagnini G, Giuffrida Stella AM, Bates TE, Clark JB (2001). Mitochondrial involvement in brain function and dysfunction: relevance to aging, neurodegenerative disorders and longevity. Neurochem Res.

[B31] Brennan WA, Bird ED, Aprille JR (1985). Regional mitochondrial respiratory activity in Huntington's disease brain. J Neurochem.

[B32] Tabrizi SJ, Workman J, Hart PE, Mangiarini L, Mahal A, Bates G, Cooper JM, Schapira AH (2000). Mitochondrial dysfunction and free radical damage in the Huntington R6/2 transgenic mouse. Ann Neurol.

[B33] Santamaria I, Alvarez-Hernandez D, Jofre R, Polo JR, Menarguez J, Cannata-Andia JB (2005). Progression of secondary hyperparathyroidism involves deregulation of genes related to DNA and RNA stability. Kidney Int.

[B34] Browne SE, Bowling AC, MacGarvey U, Baik MJ, Berger SC, Muqit MM, Bird ED, Beal MF (1997). Oxidative damage and metabolic dysfunction in Huntington's disease: selective vulnerability of the basal ganglia. Ann Neurol.

[B35] Polidori MC, Mecocci P, Browne SE, Senin U, Beal MF (1999). Oxidative damage to mitochondrial DNA in Huntington's disease parietal cortex. Neuroscience Letters.

[B36] Calkins MJ, Jakel RJ, Johnson DA, Chan K, Kan YW, Johnson JA (2005). Protection from mitochondrial complex II inhibition in vitro and in vivo by Nrf2-mediated transcription. Proc Natl Acad Sci USA.

[B37] Brouillet E, Conde F, Beal MF, Hantraye P (1999). Replicating Huntington's disease phenotype in experimental animals. Prog Neurobiol.

[B38] Benchoua A, Trioulier Y, Zala D, Gaillard MC, Lefort N, Dufour N, Saudou F, Elalouf JM, Hirsch E, Hantraye P (2006). Involvement of mitochondrial complex II defects in neuronal death produced by N-terminus fragment of mutated huntingtin. Mol Biol Cell.

[B39] Jakel RJ, Townsend JA, Kraft AD, Johnson JA (2007). Nrf2-mediated protection against 6-hydroxydopamine. Brain Research.

[B40] Andersen JK (2004). Oxidative stress in neurodegeneration: cause or consequence?. Nat Med.

