# Supplementary material for: Mutant huntingtin activates Nrf2-responsive genes and impairs dopamine synthesis in a PC12 model of Huntington's disease
Source: BMC Mol Biol. 2008 Oct 9;9:84. doi: 10.1186/1471-2199-9-84 (PMC2588454; doi:10.1186/1471-2199-9-84)
Supplement: Additional file 1 — Primer sequences for quantitative RT-PCR analysis [file 1471-2199-9-84-S1.doc]

Additonal Table 1: Primer sequences for quantitative RT-PCR analysis

| **Gene name** | **Forward/Reverse** | **5’to 3’ Sequence** |
| --- | --- | --- |
| β− Actin | For | ctggctcctagcaccatga |
|  | Rev | tagagccaccaatccacaca |
| Ywhaz | For | aaatgagctggtgcagaagg |
|  | Rev | ggctgccatgtcatcgtat |
| Tyrosine Hydroxylase | For | ggaacggtactgtggctacc |
|  | Rev | ttcaagaagcgggacacg |
| Dopamine β-hydroxylase | For | caaggattatgtcattgaggatga |
|  | Rev | gcctccagggactggaat |
| GTP cyclohydrolase 1 | For | cagtggccatcacagaagc |
|  | Rev | tgtgttgcttcaatcactaccc |
| Sepiapterin reductase | For | ggagctggtggactgtgg |
|  | Rev | gattggaaggtgtccctttg |
| Dopa decarboxylase | For | ccgtgaattccggagaag |
|  | Rev | cctcaatgccgtccagatag |
| NF-E2 related factor 2 | For | ttcctagcagagcccagtg |
|  | Rev | tcagagagcgactgactaatgg |
| NAD(P)H dehydrogenase, quinone 1 | For | tcagcgcttgacactacgat |
|  | Rev | cgtgggccaatacaatcag |
| Glutathione S-transferase, alpha 4 | For | cagttgggcagacatacagc |
|  | Rev | cagacaacacaggagcactga |
| Glutathione S-transferase, pi 2 | For | tgctggtccaccaagtcc |
|  | Rev | gagccacataggcagagagc |
| Catalase | For | agcttcagcgcaccagag |
|  | Rev | catctgcagagcactggcta |
| Glutamate-cysteine ligase, catalytic subunit | For | aagcctcctcctccaaactc |
|  | Rev | tacctccattggtcggaact |
| Glutamate-cysteine ligase, modifier subunit | For | gacattgaagcccaggagtg |
|  | Rev | gcttttcacgatgaccgagt |
| Heme oxygenase | For | tcaagcacagggtgacagaa |
|  | Rev | cagctcctcaaacagctcaa |
| Transaldolase 1 | For | acagatgcctgcctaccaag |
|  | Rev | cccagcttcttgccgtag |
| Ferritin light chain 1 | For | cagccgccttacaagtctct |
|  | Rev | ggtcctaggagccgaagg |
| Malic enzyme 1 | For | ccgtagaaacaccctttctca |
|  | Rev | ccctaacaaaaacaccccagt |
| Glutathione peroxidase 1 | For | tctgaattccctcaagtatgtcc |
|  | Rev | cattcacctcgcacttctca |
| Superoxide dismutase 1 | For | gagaggcatgttggagacct |
|  | Rev | atggacacattggccacac |
| Superoxide dismutase 2 | For | tggacaaacctgagccctaa |
|  | Rev | gcttgatagcctccagcaac |
| Metallothionein 3 | For | atgcacgaactgcaagaaga |
|  | Rev | ttggcacacttctcacatcc |
|  |  |  |
